# Prevalence of obstructive sleep apnea in patients with intracranial aneurysms

**DOI:** 10.1007/s00701-026-07012-0

**Published:** 2026-08-14

**Authors:** Riina Pirinen, Salla Ylä-Herttuala, Antti Lindgren, Nils Danner, Timo Koivisto, Juha E. Jääskeläinen, Anu Muraja, Esa Mervaala

**Affiliations:** 1 Department of Clinical Neurophysiology, Kuopio University Hospital, Puijonlaaksontie 2, 70210 Kuopio, Finland; 2 Department of Radiology, Kuopio University Hospital, Kuopio, Finland; 3 Department of Neurosurgery, Kuopio University Hospital, Kuopio, Finland; 4 Institute of Clinical Medicine, Faculty of Health Sciences, University of Eastern Finland, Kuopio, Finland

**Keywords:** Obstructive sleep apnea, Intracranial aneurysm, Respiratory polygraphy, Apnea–hypopnea index, Prevalence

## Abstract

**Purpose:**

Obstructive sleep apnea (OSA) is associated with various cardiovascular and cerebrovascular conditions; however, its prevalence among patients with intracranial aneurysms (IAs) remains insufficiently characterized. Emerging evidence suggests that OSA may contribute to IA formation, progression, and outcomes after rupture.

**Methods:**

This study included 244 participants: 84 IA patients without prior clinical suspicion of OSA and 160 OSA-symptomatic comparators. Overnight respiratory polygraphy (RP) was used to assess apnea–hypopnea index (AHI) and presence of OSA. Group differences were analyzed using models adjusted for age, BMI, and sex.

**Results:**

Among IA patients, 50.0% had findings consistent with OSA on RP (AHI ≥ 5 events per hour), of whom 27.4% were classified as mild, 15.5% as moderate, and 7.1% as severe. The corresponding proportions in the OSA-symptomatic comparison group were 71.3% for overall OSA, and 36.3%, 18.1%, and 16.9%, for mild, moderate, and severe, respectively. Log-transformed AHI in IA patients did not differ significantly from that in the symptomatic comparison group (F(1,238) = 1.88, *p* = 0.171, partial η^2^ = 0.008).

**Conclusion:**

RP findings demonstrated a high and largely unrecognized burden of OSA in IA patients. These findings support more systematic and proactive screening for OSA in IA patients, particularly among those with established OSA risk factors.

## Introduction

Obstructive sleep apnea (OSA) is a common and often underdiagnosed sleep-related breathing disorder characterized by recurrent upper airway obstruction during sleep, resulting in intermittent hypoxemia, autonomic fluctuation, and sleep fragmentation [3, 22]. Globally, OSA affects an estimated 9%–38% of the adult population, with higher prevalence in older and obese individuals, and its prevalence appears to be increasing over time, likely driven by rising rates of obesity and aging populations [20]. The prevalence of moderate-to-severe OSA has been estimated to be as high as approximately 17% among middle-aged men (50–70 years old) and 9% among women of the same age group [14]. In Finnish population-based studies, the prevalence of clinically diagnosed OSA has been reported to be 3.7%–4.2% [12]. Established risk factors for OSA include obesity, older age, male sex, large neck circumference, smoking, alcohol consumption, nasal congestion, and certain genetic or craniofacial characteristics [9, 24, 27, 30]. OSA has also been associated with various cardiovascular and cerebrovascular conditions, including hypertension and stroke [4, 19, 23, 29]. The prevalence of OSA is markedly increased in these patient populations [1, 13, 28].

Intracranial aneurysms (IAs) are localized dilations of cerebral arteries caused by weakening of the vessel wall and are relatively common, with a prevalence of approximately 3%–5% in the general population [5, 6, 25]. They exhibit variable morphology, with saccular aneurysms representing the most common type [15]. Although many aneurysms remain asymptomatic, their rupture may result in subarachnoid hemorrhage, a life-threatening condition associated with substantial morbidity and mortality [2, 21]. Various risk factors for aneurysm formation and rupture have been identified, including smoking, hypertension, and genetic predisposition [10, 26].

OSA and unruptured IAs share common risk factors, including smoking, alcohol intake, and hypertension [8] (Table 1). Increasing evidence suggests that OSA may also contribute to the development and progression of IAs [7, 8, 31]. A recent study showed that IAs are more prevalent in patients with OSA [8], while another study suggested that OSA is also more prevalent in patients with IAs [31]. OSA has also been associated with unfavorable outcomes in patients with ruptured IAs, including larger aneurysm size, increased vasospasm, and poor functional outcomes [7]. These observations indicate that OSA may be a modifiable risk factor influencing IA progression and clinical outcomes. Despite these findings, the prevalence of OSA in the IA patient population remains insufficiently characterized.

**Table 1:** Common risk factors for obstructive sleep apnea and intracranial aneurysm

Risk Factor	OSA	IA	Notes
Age	Yes	Yes	Risk increases with age
Sex	Male predominance	Female predominance	Different sex distribution
Hypertension	Yes	Yes	Major shared vascular risk factor
Smoking	Yes	Yes	Strong modifiable risk factor
Alcohol consumption	Yes	Yes	Associated with severity and rupture risk
Obesity	Yes (major risk factor)	Indirect	Via metabolic and vascular effects
Genetic predisposition	Possible	Yes	Family history relevant
Dyslipidemia/metabolic syndrome	Yes	Yes	Cardiovascular risk cluster

Abbreviations: *OSA* obstructive sleep apnea, *IA* intracranial aneurysm

Against this background, the aims of this study were to determine the prevalence of undiagnosed OSA in patients with IAs and to compare OSA-related findings between IA patients and OSA-symptomatic comparators in the Eastern Finnish catchment population of Kuopio University Hospital (KUH).

## Methods

### Study population

Our study was conducted after approval by the Regional Ethics Committee of Eastern Finland (approval number 139/13.02.00/2017). Patients were recruited from the well-characterized KUH Aneurysm Registry, maintained by the Department of Neurosurgery, the sole provider of both acute and elective IA treatment in its geographical catchment area in Eastern Finland, serving a population of approximately 850 000 inhabitants. Eligible participants from the registry were adult patients with at least one IA confirmed by angiographic imaging (computed tomography angiography, magnetic resonance angiography, or digital subtraction angiography). Participants were contacted either by post or during an outpatient clinic visit and invited to participate in the study.

The prespecified inclusion criteria for the study were age between 18 and 65 years and sufficient functional and cognitive ability to complete overnight respiratory polygraphy (RP). Patients with a history of ischemic stroke and those unable to provide informed consent were excluded. Participants provided written informed consent prior to study participation.

The comparison group was retrospectively collected from an existing database of the Department of Clinical Neurophysiology at KUH. The ratio of IA patients to symptomatic comparators was set to achieve 1:2. The inclusion criterion was age between 34 and 65 years, matching the age range of the gathered IA group. Comparators had originally been referred for RP due to symptoms suggestive of OSA. The presence of IAs was not systematically assessed in the comparison group.

### Data acquisition

Participants in the IA group underwent overnight RP using a type III portable sleep apnea monitoring device (Embletta PDS; Medcare, Reykjavik, Iceland). Recordings were performed between September 2017 and April 2018. RP recordings for the comparison group, performed between April and December 2025, using a type III portable sleep apnea monitoring device (Nox T3; Nox Medical, Inc., Reykjavik, Iceland), were retrieved from the registry.

RP recordings were performed predominantly at home, and each RP had a diagnostic setup that included continuous measurement of thoracic and abdominal respiratory effort by impedance plethysmography, body position monitored by a position sensor, leg EMG electrodes, oxygen saturation and pulse rate measured by pulse oximetry, and nasal airflow recorded using a pressure cannula and thermistor. Recording time was adjusted according to each participant’s usual sleep schedule. Basic demographic and clinical data were collected at the time of RP recording.

### Data processing

RP recordings were initially analyzed using automated scoring and subsequently reviewed and corrected manually by trained personnel according to the American Academy of Sleep Medicine (AASM) scoring guidelines. Apnea was defined as a ≥ 90% reduction in airflow lasting at least 10 s, whereas hypopnea was defined as a ≥ 30% reduction in airflow lasting at least 10 s and associated with a ≥ 3% decrease in oxygen saturation.

AHI was calculated as the total number of apnea and hypopnea events per estimated hour of sleep. Obstructive apneas were defined as respiratory events occurring in the presence of continued respiratory effort, whereas central apneas were characterized by the absence of respiratory effort during the event. An AHI < 5 events per hour was classified as normal, 5–14.9 as mild, 15–29.9 as moderate, and ≥ 30 as severe OSA.

### Statistical analysis

Various statistical analyses were performed. Basic descriptive statistics were calculated. Due to the skewed distribution of AHI, logarithmic transformation was applied prior to analysis (logAHI). Group differences in logAHI were analyzed using analysis of covariance (ANCOVA), with group status (IA vs. comparator) and sex included as fixed factors and age and BMI as covariates.

Differences in OSA severity between the groups were evaluated using ordinal logistic regression, and the presence of OSA was analyzed using binary logistic regression, both adjusting for sex, age, and BMI. Age was categorized into two groups (34–49 and 50–65 years), BMI into three groups according to World Health Organization (WHO) criteria (normal weight < 25 kg/m^2^, overweight 25–29.9 kg/m^2^, and obesity ≥ 30 kg/m^2^), and AHI into three categories: normal (< 5 events/h), mild OSA (5–14.9 events/h), and moderate-to-severe OSA (≥ 15 events/h). Moderate and severe OSA categories were subsequently combined into a single category to ensure sufficient statistical power.

As sensitivity analyses, the ANCOVA was repeated using categorized age and BMI, and the ordinal logistic regression was repeated using age and BMI as continuous covariates.

Statistical significance was defined as a two-sided p-value < 0.05. All analyses were performed using IBM SPSS Statistics (version 29; IBM Corp., Armonk, NY, USA).

## Results

The final study population consisted of 244 adults, including 84 patients with IAs without prior clinical suspicion of OSA and 160 symptomatic comparators. Baseline characteristics of the study population are presented in Table 2. Compared with symptomatic comparators, IA patients were older, had a lower BMI, and were less frequently male (all *p* < 0.05).

**Table 2:** Baseline characteristics and presence/severity of obstructive sleep apnea in study groups

Variable	IA patients (*n* = 84)	Comparison group (*n* = 160)	*p*-value
Age, mean (SD), years	55.1 (7.7)	49.0 (9.1)	< 0.001
BMI, mean (SD), kg/m^2^	27.0 (4.6)	32.0 (6.8)	< 0.001
Male sex, n (%)	34 (40.5)	87 (54.4)	0.039^a^
AHI, mean (SD), events/h	11.3 (15.0)	16.4 (18.5)	0.022
Normal (AHI < 5), n (%)	42 (50.0)	46 (28.7)	0.044^b^
Mild OSA (AHI 5–14.9), n (%)	23 (27.4)	58 (36.3)	
Moderate OSA (AHI 15–29.9), n (%)	13 (15.5)	29 (18.1)	
Severe OSA (AHI ≥ 30), n (%)	6 (7.1)	27 (16.9)	

Abbreviations: *AHI* apnea–hypopnea index, *BMI* body mass index, *OSA* obstructive sleep apnea, *IA* intracranial aneurysm, ^a^ Pearson χ^2^ test for sex distribution, ^b^ overall comparison of OSA severity distribution between groups derived from ordinal logistic regression analysis

**Fig. 1:**
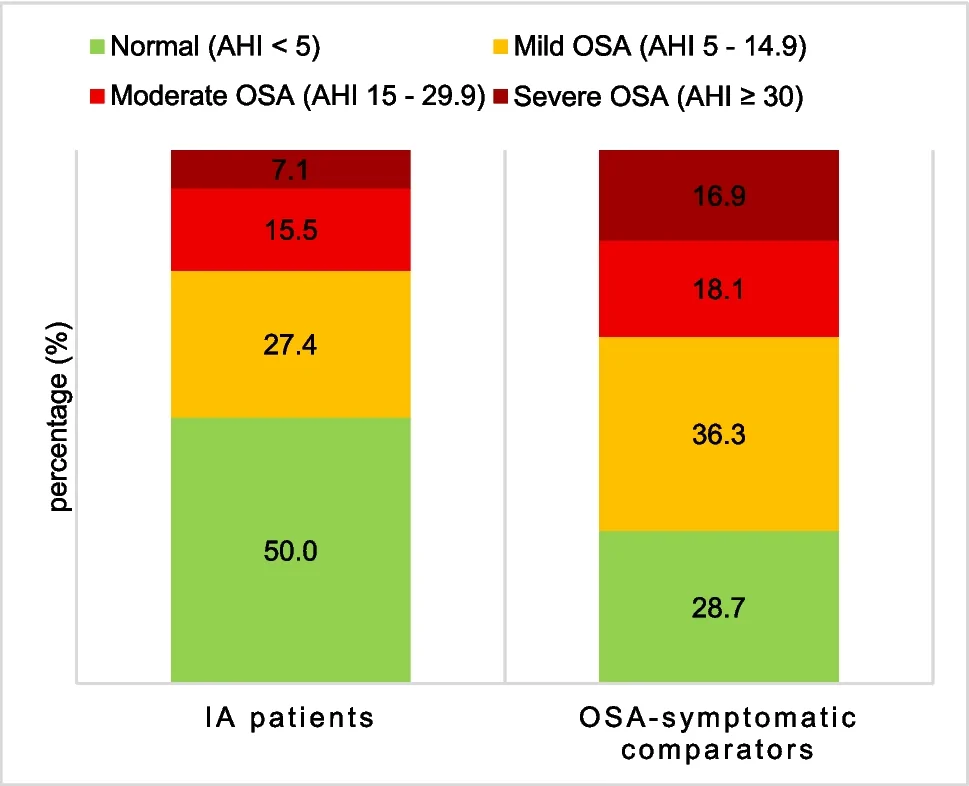
Distribution of obstructive sleep apnea (OSA) finding by group

The crude mean AHI was lower in the IA group than in the comparison group (11.3 vs. 16.4 events/h, *p* = 0.022). Overall, 50.0% of IA patients had RP findings consistent with OSA, compared with 71.3% of symptomatic comparators. The distribution of OSA presence and severity is presented in Table 2 and Fig. 1. In adjusted analyses, OSA prevalence remained higher in the OSA-symptomatic comparison group than in the IA group (OR 2.56, 95% CI 1.19–5.26, *p* = 0.015).

ANCOVA was performed using log-transformed AHI (logAHI), the results of which are presented in Table 3. Higher BMI, male sex, and increasing age were all independently associated with higher logAHI (all *p* < 0.001), with BMI showing the strongest association, followed by sex and age. In contrast, IA presence was not significantly associated with logAHI (F(1,238) = 1.88, *p* = 0.171, partial η^2^ = 0.008). No significant interaction between IA and sex was observed. The overall model was statistically significant and explained 38.6% of the variance in logAHI.

**Table 3:** ANCOVA factors associated with apnea–hypopnea index

Source	B	df	F	*p*-value	Partial η^2^
Age	0.039	1	43.04	< 0.001	0.153
BMI	0.068	1	65.84	< 0.001	0.217
Sex	−0.779	1	48.46	< 0.001	0.169
Group (IA vs. comparators)	0.141	1	1.88	0.171	0.008
Group × sex	0.047	1	0.05	0.828	0.000
Error	-	238	–	–	–

Model statistics: F(5,238) = 29.93, p < 0.001; R^2^ = 0.386; adjusted R^2^ = 0.373

Abbreviations: *ANCOVA* analysis of covariance (model adjusted for age, BMI, and sex), *B* unstandardized regression coefficient (log-transformed AHI), *df* degrees of freedom, *F* F-statistic, *η*^2^ partial eta squared, *AHI* apnea–hypopnea index, *BMI* body mass index

Ordinal logistic regression analysis showed higher OSA severity in the OSA-symptomatic comparison group than in the IA group (OR 1.86, 95% CI 1.02–3.42, *p* = 0.044; Table 4). Increasing age, obesity, and male sex were also independently associated with greater OSA severity. The proportional odds assumption was satisfied (*p* = 0.353), supporting the validity of the model.

**Table 4:** Ordinal logistic regression analysis of factors associated with severity of obstructive sleep apnea

Variable	β	OR (95% CI)	*p*-value
Age 34–49 vs. 50–65	− 1.735	0.18 (0.10–0.32)	< 0.001
Normal weight vs. obese	− 2.206	0.11 (0.05–0.25)	< 0.001
Overweight vs. obese	− 1.038	0.35 (0.20–0.64)	< 0.001
Female vs. male	− 1.961	0.14 (0.08–0.25)	< 0.001
Comparators vs. IA	0.623	1.86 (1.02–3.42)	0.044

Abbreviations: β regression coefficient, *OR (95% CI)* odds ratio with 95% confidence interval, *IA* intracranial aneurysm

Sensitivity analyses using categorized age and BMI in the ANCOVA and continuous age and BMI in the ordinal logistic regression yielded results consistent with the primary analyses.

## Discussion

In this study, OSA was highly prevalent among IA patients despite the absence of prior clinical suspicion, with half of the patients showing RP findings suggestive of OSA and nearly one-quarter having moderate-to-severe disease. This does not necessarily indicate that patients were asymptomatic, but rather that symptoms may have been mild or nonspecific (e.g., snoring and daytime sleepiness), attributed to normal aging, and insufficient to prompt clinical evaluation or healthcare seeking. Lack of systematic screening and limited clinical awareness may also have contributed to such underdiagnosis. These findings highlight OSA as a common and likely underdiagnosed comorbidity in this patient population.

These findings should be interpreted in the context of existing literature suggesting a possible association between OSA and IAs. Jung et al. reported in a polysomnography (PSG)-based study that IAs were more prevalent among patients with OSA than in those without it (12.1% vs. 5.9%, *p* = 0.031), and that OSA remained independently associated with aneurysm presence after adjustment for confounders. In the same study, patients with OSA also had larger aneurysms, supporting a possible role of OSA in aneurysm development and progression [8]. Similarly, Zaremba et al. reported a 42.6% prevalence of OSA among patients with IAs and found that OSA was associated with larger aneurysm size and increased use of antihypertensive medication. They also reported that all IA patients with OSA exhibited at least one clinical symptom of OSA, with snoring and witnessed apnea being the most common. The authors further suggested that OSA may contribute to accelerated aneurysm growth [31]. In addition, Chernyshev et al. showed that OSA was associated with an increased risk of vasospasm and poor functional outcomes in patients with ruptured IAs (RIAs) and reported that the symptom-based prevalence of OSA in patients with RIAs was five times higher than in non-aneurysm patients [7]. Taken together, these findings suggest that OSA is a clinically relevant and potentially modifiable comorbidity in patients with IAs. Further research is warranted to better understand the role of OSA in aneurysm formation, progression, and outcomes, and to determine whether improved recognition and treatment of OSA may translate into improved IA patient outcomes.

We previously showed that obstructive apneas in severe OSA induce rapid and pronounced peri-apneic hemodynamic changes [11]. Studies by Riedel et al. have provided advanced understanding of intracranial dynamics related to respiratory events [16–18]. They demonstrated that respiratory events were consistently associated with transient intracranial pressure (ICP) elevations (B-waves), with larger ramp-type elevations occurring during rapid eye movement (REM) sleep and smaller sinusoidal elevations during non-REM (NREM) sleep [16, 18]. In a small subgroup of patients, continuous positive airway pressure (CPAP) treatment also reduced the frequency of transient ICP elevations [18]. Their subsequent experimental study further demonstrated that these transient ICP elevations were driven primarily by negative intrathoracic pressure-induced elevations in central venous pressure and impaired cerebral venous outflow, while hypercapnia further augmented the response [17]. These observations provide a plausible hemodynamic context for how respiratory events in OSA may repeatedly stress the cerebral arterial wall.

As OSA has been associated with adverse aneurysm-related outcomes and may represent a modifiable risk factor, early identification of OSA may be clinically relevant. However, further evidence is needed to determine the optimal screening strategy, in terms of whether universal screening for OSA or a more targeted approach focusing on IA patients with established OSA risk factors is more appropriate. Clinicians should be aware of the possibility of OSA in patients with IAs and maintain a low threshold for diagnostic evaluations.

A few limitations of this study should be acknowledged. AHI data from asymptomatic population-based cohorts are limited, particularly within the studied age range, as most population-based studies rely on symptom-based assessments rather than objective AHI measurements obtained by RP or PSG. Consequently, a comparison with OSA-symptomatic comparators was considered necessary, as it enabled comparison against a clinically relevant population undergoing RP evaluation in routine practice. However, the use of a symptom-enriched comparison group likely introduced substantial selection bias that necessarily attenuated the differences between the two groups. Had the IA group been compared with a truly asymptomatic population-based control group, the observed odds of OSA among IA patients would likely have been significantly higher. The presence of IAs was not systematically assessed in the comparison group, as this was not part of the study protocol. As a result, the possibility of undiagnosed IAs among comparators cannot be ruled out, which may have led to a more conservative estimate of the association. The exclusion of patients with a history of ischemic stroke from the IA group may have contributed to a lower observed prevalence of OSA, as OSA prevalence exceeds 60% among stroke patients [28]. Moreover, owing to the limited number of moderate and severe OSA cases and sparse data in some combinations of covariates, these categories were combined in the ordinal logistic regression analysis to ensure reliable estimation. This may have limited the ability to detect differences across the full spectrum of OSA severity, particularly those specific to severe OSA (AHI ≥ 30 events/h). In addition, the continuous ANCOVA analysis of logAHI did not demonstrate an independent association between study group and AHI, whereas the binary and ordinal logistic regression analyses identified significant between-group differences in OSA presence and severity. Sensitivity analyses using categorized age and BMI in the ANCOVA and continuous age and BMI in the ordinal logistic regression yielded results consistent with the primary analyses. The discrepancy therefore most likely reflects differences in the statistical hypotheses tested rather than the categorization of these covariates. Accordingly, the categorical findings should be interpreted in the context of the non-significant continuous analysis. RPs for comparators were performed in 2025 and for IA patients in 2017–2018, resulting in a time difference of 7–8 years. Temporal changes in population characteristics, such as increasing age, obesity prevalence, clinical awareness of OSA, or referral practices, may theoretically have influenced the findings. However, the analyses were adjusted for major demographic confounders, including age, sex, and BMI. Finally, RP recordings in the IA and comparison groups were performed using devices from different manufacturers. However, both systems were level III portable monitoring devices in accordance with the American Academy of Sleep Medicine (AASM) recommendations, recording comparable cardiorespiratory signals using similar measurement principles. Therefore, this manufacturer-related difference is unlikely to have materially affected AHI values.

Further analyses focusing on associations between specific IA characteristics and OSA findings within our IA study population are ongoing and will be presented in future work.

## Conclusion

Polygraphic evidence demonstrating a high prevalence of OSA in IA patients highlights a substantial and largely unrecognized burden of OSA in this patient population. Given the potential impact of OSA on aneurysm progression and clinical outcomes, increased clinical awareness and a low threshold for diagnostic evaluation are warranted. Further studies are needed to clarify the relationship between OSA and IA and to determine the potential benefits of more systematic OSA screening and treatment as part of IA patient care.

## Data Availability

The data supporting this study are available from the corresponding author upon reasonable request.
